# Arrhythmogenic Cardiomyopathy *PKP2*-Related: Clinical and Functional Characterization of a Pathogenic Variant Detected in Two Italian Families

**DOI:** 10.3390/genes16040419

**Published:** 2025-03-31

**Authors:** Enrica Marchionni, Sonia Lomuscio, Andrea Latini, Michela Murdocca, Fabiana Romeo, Cinzia Crescenzi, Leonardo Calò, Giuseppe Novelli, Ruggiero Mango, Federica Sangiuolo

**Affiliations:** 1 Medical Genetics Unit, Tor Vergata University Hospital, 00133 Rome, Italy; enrica.marchionni@ptvonline.it (E.M.); sonialomuscio@libero.it (S.L.); novelli@med.uniroma2.it (G.N.); 2 Department of Biomedicine and Prevention, University of Rome Tor Vergata, 00133 Rome, Italy; a.latini@med.uniroma2.it (A.L.); michela.murdocca@uniroma2.it (M.M.); 3 UniCamillus, Saint Camillus International University of Health Sciences, 00131 Rome, Italy; 4 Division of Cardiology, Policlinico Casilino, 00169 Rome, Italy; fabiana.romeo87@gmail.com (F.R.); crescenzi.cinzia@gmail.com (C.C.); leonardo.calo@tin.it (L.C.); 5 Cardiology Unit, Department of Emergency and Critical Care, Policlinico Tor Vergata, 00131 Rome, Italy

**Keywords:** *PKP2*, Arrhythmogenic Cardiomyopathy, Sudden Cardiac Death

## Abstract

**Background/Objectives:** *PKP2* (MIM *602861) is the most commonly gene associated with Arrhythmogenic Cardiomyopathy (ACM), an inherited cardiac muscle disorder. The aim of this study was to characterize the phenotypical effect of a heterozygous pathogenic c.2443_2448delAACACCinsGAAA variant in *PKP2* gene (NM_004572), detected in two Italian families. **Methods**: Next Generation Sequencing (NGS) analysis was carried out on two probands, testing a multigenic targeted panel. Segregation analysis through Sanger sequencing detected other three and six positive members, in Family 1 and 2, respectively. Thus, eleven positive patients were identified overall. A deep clinical evaluation was performed according to age groups and clinical parameters (symptoms, electrocardiogram, imaging, and devices). To investigate the molecular effect of the identified variant on *PKP2* expression level, total RNA was isolated from peripheral blood mononuclear cells (PBMCs) and quantitative RT-polymerase chain reaction was performed. PKP2 expression at the protein level was analyzed on PBMCs by Western blot analysis. **Results**: *PKP2* transcriptional levels resulted to be reduced by 48% in cells carrying c.2443_2448delAACACCinsGAAA variant compared to WT cells (*p* = 0.00015). Importantly, Western blot confirmed the reduced level of PKP2 protein in two heterozygous carriers of the variant, confirming the haploinsufficiency effect. **Conclusions**: The clinical onset of ACM can be Sudden Cardiac Death, and hence, it is recommended to perform a segregation test on first-degree relatives of pathogenic variant carriers, even if they are asymptomatic, with the purpose of promptly detecting those at risk.

## 1. Introduction

Arrhythmogenic Cardiomyopathy (ACM, MIM *609040) is a genetic disease characterized by incomplete penetrance and a wide variable expressivity. In most cases, it is caused by pathogenic variants in genes encoding for desmosomal proteins, which are involved in cardiomyocyte adhesion [1]. Among the desmosomal genes, *Plakophilin-2* (*PKP2*, MIM *602861) is the most frequently associated with ACM [2]. Reduced expression of desmosomal proteins has been shown to induce cardiac dysfunction [3]. This is attributable to a progressive weakening of cell-to-cell junctions leading to fibrotic remodeling areas, due to a decrease in mechanical traction tolerance [4].

The consequential cardiomyocyte apoptosis results in an uneven slowing of signal propagation, which implies the development of arrhythmias in affected patients. This explains why ACM is associated with a high risk of Sudden Cardiac Death (SCD), especially when *PKP2* mutation carriers have not undergone appropriate cardiological follow-up [5].

Clinical diagnosis of ACM is based on evaluating signs and symptoms, imaging, histological characterization, and electrocardiographic features. It is also important to obtain medical information about patients’ pedigrees, as approximately 30% to 50% of those affected have a positive family history [6].

According to the *2010 Revised Task Force Criteria for the Diagnosis of Arrhythmogenic Cardiomyopathy* (formerly known as Arrhythmogenic Right Ventricular Cardiomyopathy/Dysplasia)*,* all the clinical features detected through the cardiological assessment can be sorted into six different categories, each consisting of major and minor criteria:

1. Morphofunctional Alterations (right ventricular (RV) dilatation or wall motion abnormalities);
2. Myocardium Histology (fibro-adipose replacement);
3. Repolarization Abnormalities (inverted T waves in right precordial leads);
4. Depolarization/Conduction Abnormalities (ε waves in the right precordial leads, prolonged QRS duration);
5. Arrhythmias (ventricular tachycardia, ventricular extrasystoles);
6. Family History (positive for ACM and/or SCD).

To confirm a clinical diagnosis of ACM it is necessary to recognize two major criteria from different categories, one major and two minor criteria from different categories, or four minor criteria from different categories [7].

Ten years after the publication of the *2010 Task Force Criteria*, the sensitivity and specificity of these diagnostic guidelines have been improved by the issuance of the *Padua criteria* [8].

To better understand the dynamics that subtend the heterogeneous expression of the disease, we investigated the clinical phenotype and evolution of eleven heterozygous carriers of the pathogenic c.2443_2448delAACACCinsGAAA variant in the *PKP2* gene (NM_004572), belonging to two different unrelated Italian families. The variant has been previously reported in literature in two patients from international cohorts of unknown geographic origin [9,10].

## 2. Case Description

Two unrelated patients were referred to our Genetics Unit for genetic counseling: Proband 1 was a 61-year-old Caucasian male (II:2), presenting a first episode of Paroxysmal Supraventricular Tachycardia (PSVT) at age 26, with ε waves in the right precordial leads (V1–V3) and right ventricular dilatation with hypokinesia on imaging. One year later, he was admitted to the emergency room for wide QRS tachycardia with left bundle branch block pattern and a synchronized direct current cardioversion was performed. Proband 2 (IV:4) was a 48-year-old Caucasian male with RV exuberant moderator band on echocardiography. Both probands had a positive family history of SCD. After obtaining informed consent for the genetic analysis, Next Generation Sequencing (NGS) was performed on genomic DNA extracted from probands’ peripheral blood mononuclear cells (PBMCs), testing a targeted panel which included genes associated with ACM and Dilated Cardiomyopathy (DCM) [genes analyzed: *ACTC1* (NM_005159), *ACTN2* (NM_001103), *BAG3* (NM_004281), *CAV3* (NM_001234), *DES* (NM_001927), *DSC2* (NM_024422), *DSG2* (NM_001943), *DSP* (NM_004415), *FLNC* (NM_001458), *JUP* (NM_002230), *LMNA* (NM_170707), *MYBPC3* (NM_000256), *MYH7* (NM_000257), *PKP2* (NM_004572), *PLN* (NM_002667), *RBM20* (NM_001134363), *SCN5A* (NM_001099404), *TNNC1* (NM_003280), *TNNI3* (NM_000363), *TNNT2* (NM_001276347), *TPM1* (NM_001018006), *TTN* (NM_001267550)].

The heterozygous c.2443_2448delAACACCinsGAAA (NM_004572) pathogenic variant in *PKP2* gene (MIM *602861) was identified in both patients.

Given the molecular results, the two probands and all at-risk relatives (according to the cascade genetic testing approach) were recommended for a cardiological check-up and genetic counseling was offered to family members to propose segregation analysis. After collecting all informed consents, Sanger sequencing was performed on the PBMCs DNA of at-risk relatives who contacted our hospital, to detect the possible presence of the above-mentioned variant.

**Figure 1:**
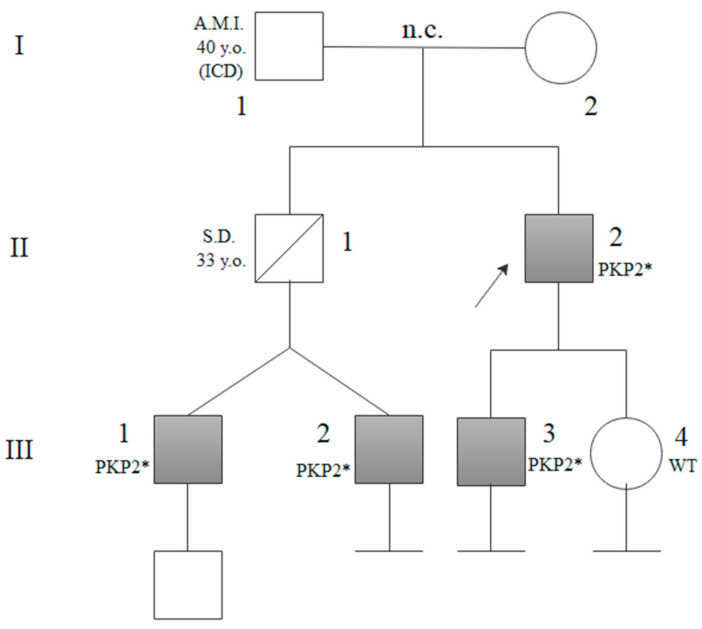
Family 1 pedigree: It was reported that the proband’s father (I:1) had an acute myocardial infarction (AMI) at the age of 40 years and then he was implanted with an implantable cardioverter defibrillator (ICD). The proband’s brother (II:1, obligate carrier of the variant) died of Sudden Death (SD) at the age of 33. All tested heterozygous carriers of the *PKP2* (NM_004572) variant (II:2; III:1; III:2; III:3) are shown in grey and are indicated with PKP2* symbol. Segregation analysis resulted negative in patient III:4, Wild-Type (WT).

Three relatives of proband 1 (Family 1; II:2) tested positive on segregation analysis (Figure 1; III:1; III:2; III:3). They were two 34-year-old twins, both professional athletes, and their 25-year-old first-degree cousin, all three asymptomatic (III:1; III:2; III:3). At the time of our consultation, one of the twins (III:2) had no alterations on instrumental examinations, whereas the other (III:1) had a mild biventricular dilatation on imaging, and cardiac magnetic resonance imaging (CMRI) identified intramyocardial LGE distributed along the inferior wall and inferoseptal mid segments of the left ventricle (LV) (Table 1).

Their cousin (III:3) had a slight change in repolarization on the electrocardiogram (EKG). His asymptomatic sister (Family 1; III:4) tested negative on segregation analysis (Figure 1).

Exercise stress testing of proband 2 (Family 2; IV:4) at 52 years showed a Premature Ventricular Contraction (PVC) during stress testing and two PVCs during recovery phase.

**Figure 2:**
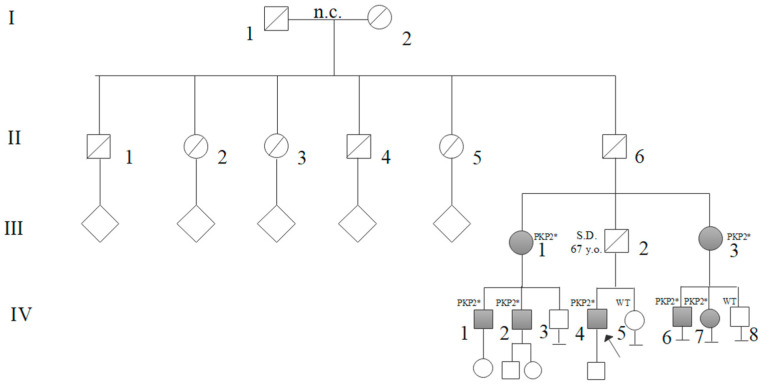
Family 2 pedigree: The proband’s father (III:2, obligate carrier of the variant) died at the age of 67 years due to Sudden Death (SD). All tested carriers of the *PKP2* (NM_004572) variant (III:1; III:3; IV:1; IV:2; IV:4; IV:6; IV:7) are shown in grey and are indicated with PKP2* symbol. Segregation analysis resulted negative in patient IV:5 and IV:8, Wild-Type (WT). n.c.: non-consanguineous.

Six relatives of proband 2 (Family 2; IV:4) tested positive on segregation analysis (Figure 2; III:1; III:3; IV:1; IV:2; IV:6; IV:7). A 70-year-old aunt (III:1) reported palpitations and a history of syncopal episodes during her youth. EKG showed T-wave inversions in the right precordial leads and echocardiography revealed the development of RV dysfunction. Exercise stress testing of III:1, at 65 years, showed PVCs, both singles and organized in couples at rest and during recovery phase. CMRI in III:1 identified subepicardial LGE distributed along basal inferolateral LV and RV walls (Table 1). She also underwent an implantable cardioverter defibrillator (ICD) insertion. Her two sons (IV:1; IV:2), i.e., first-degree cousins of proband 2, were asymptomatic. IV:2 had RV dilation and dysfunction at 46 years; exercise stress testing at 40 years showed monomorphic Non-sustained Ventricular Tachycardia at a rate of 10 beats per minute during the recovery phase, and at 43 years, several PVCs were evident at rest and during the recovery phase. He had an implantable loop recorder (ILR) insertion. The other cousin (IV:1) presented ventricular repolarization phase anomalies at 42 years. An aunt of 72 years old (III:3) presented palpitations, T-wave inversions in the right precordial leads, ε waves, and RV dilation and dysfunction. CMRI identified intramyocardial LGE of the inferoseptal basal LV wall. She also had an ILR insertion. Moreover, her 50-years son (IV:6) and 52-years daughter (IV:7) tested positive at segregation analysis (Figure 2). Both were asymptomatic at the time of our consultation. IV:6, at 51 years, showed several PVCs during all the exercise testing. IV:7 had low-voltage QRS in limb leads on the EKG, and at 53 years, showed a PVC in couple during exercise stress testing. In total, eleven positive patients were identified.

## 3. Materials and Methods

### 3.1. Clinical Evaluation

One of the aims of our study was to define a prognostic profile of ACM associated with *PKP2* c.2443_2448delAACACCinsGAAA pathogenic variant. The highly variable phenotypic expression observed in our patient cohort led us to search for objective parameters, to predict the course of the disease.

Therefore, we collected clinical data, according to the major and minor diagnostic criteria mentioned above [7]. Then, we categorized our patients into age groups with the aim of characterizing the evolution of ACM (Table 1).

### 3.2. Genetic Analysis

The DNA was extracted from PBMCs of probands (Family 1; II:2 and Family 2, IV:4) and their relatives with the EZ1 DNA Blood kit (Qiagen, Hilden, Germany) following the manufacturer’s protocol. DNA was analyzed by Nanodrop (Thermofisher, Waltham, MA, USA) to evaluate the quality by measurement of the levels of absorbance at different wavelengths. At the same time, the samples were quantified with the Qubit Fluorometer 2.0 using the Qubit High Sensitivity Assay Kit (Thermofisher, Waltham, MA, USA) to determine concentration. An on-demand custom panel was designed using Ion AmpliSeq Designer software 7.48 version, selecting 22 genes following the international recommendations associated with Cardiomyopathies [11]. To generate the libraries, 15 ng of gDNA were necessary for each sample, using the Ion Chef System (Thermofisher, Waltham, MA, USA) and following the manufacturer’s instructions. The resultant template of samples was sequenced on the Ion Torrent S5 platform (Thermofisher, Waltham, MA, USA) using the 530 chip. Alignment and variant calling were carried out by the Torrent Suite (Thermofisher, Waltham, MA, USA) using as human reference genome the GRCh37/hg19 version. Data were analyzed using the IonReporter software (version 5.16.1), and the pathogenicity of the identified variants was evaluated according to the American College of Medical Genetics and Genomics (ACMG) standards and guidelines [12]. Sanger sequencing was performed for confirmation in the probands and segregation analysis.

### 3.3. PKP2 Gene Expression in PBMCs

Total RNA was extracted from PBMCs from three *PKP2* mutation carriers (Family 1; II:2; III:1; Family 2; III:1) and two age-matched controls using the TRIzol reagent (Ambion, Carlsbad, CA, USA) standard protocol. RNA quality and concentration were evaluated by the NanoDrop ND-1000 Spectrophotometer (Thermo Fisher Scientific, Waltham, MA, USA) and RNA integrity was assessed by standard denaturing agarose gel electrophoresis. Reverse transcription of 1 μg of RNA was performed using High-Capacity cDNA Reverse Transcription Kit (Applied Biosystems, Waltham, MA, USA). Expression analysis was performed by quantitative RT-polymerase chain reaction (RT-qPCR) (SYBR Green Assay, Applied Biosystems), using the 7500 Real-Time PCR System (Applied Biosystems, Waltham, MA, USA). The primers used to detect gene expression are: Fw-AAGATGACCCAGATCATGTTTGAGACC and Rv-AGCCAGGTCCAGACGCAGGAT for β-Actin; Fw-AGCTCGGAAGAGGGTTAACCA and Rv-AGCCGAGGTACCCCATTTAGTT for PKP2. Each sample was analyzed in triplicate and, to standardize the results, each assay was run with an endogenous control (β-Actin). The 2-DCt method was used to quantify the relative gene expression levels.

### 3.4. PKP2 Protein Expression in PBMCs

PBMCs were lysed in RIPA-Buffer (PierceTM RIPA Buffer, REF 89901) plus a cocktail of protease inhibitors (HaltTM Protease Inhibitor Cocktail 100X, Prod. #1862209, Thermo Fisher Scientific). The protein concentration was determined using the Bradford method. In total, 50 μg of proteins were separated by 4-12%SDS-PAGE gel (NW04125BOX, Thermo Fisher Scientific), then transferred into a nitrocellulose membrane (88018, Thermo Fisher Scientific). The primary antibodies used are: anti-PKP2 antibody (PA5-75421, Thermo Fisher Scientific) and GAPDH, (MA5-15738, Thermo Fisher Scientific). Proteins were visualized with Clarity Max™ Western ECL Substrate (1705062, Biorad, Hercules, CA, USA), and chemiluminescence signals were detected using the Image Quant LAS 4000 mini.

### 3.5. Statistical Analysis

Molecular experiments were performed in technical triplicates and data were analyzed using GraphPad Prism 8. The difference between groups was tested by one-way ANOVA test. Data are expressed in arbitrary units, normalized to the Wild-Type condition, which is set to 1. Statistical significance was established at ** *p* < 0.01.

## 4. Results

### 4.1. Molecular Results

NGS analysis in probands II:2 from Family 1 and IV:4 from Family 2 detected the c.2443_2448delAACACCinsGAAA *PKP2* (NM_004572) heterozygous variant. The variant is a frameshift mutation located in exon 12 that changes the reading frame and produces an aberrant mRNA and a truncated or absent protein, due to mRNA decay. We found an unknown frequency in GnomAD (v.4.1) and ClinVar databases. The variant was reported in two previously published cohorts [9,10]. Another variant, consisting in a deletion, c.2447_2448del, and causing the same reading frame change, has been previously reported in patients with ACM in two different studies [13,14]. Subsequent segregation analysis through Sanger identified the variant in heterozygosity in three members of the first family and six members of the second family (Figure 1 and Figure 2). For variant classification we considered the following criteria from the ACMG consensus [12]:

- PVS1: Null variant in a gene where LOF is a known mechanism of disease;
- PM2: Absent from controls (or at extremely low frequency if recessive) in Exome Sequencing Project, 1000 Genomes Project, or Exome Aggregation Consortium;
- PP1: Cosegregation with disease in multiple affected family members in a gene definitively known to cause the disease.

Thus, we classified this variant as pathogenic, according to the ACMG variant classification.

### 4.2. Clinical Characterization

As expected, no symptoms (palpitations and/or syncope) were reported from the age of 20 up to the age of 40. An inverted isodiphasic T wave on precordial leads v4–v6 was observed in the only patient in the 20–30 age group. Early structural remodeling (mild biventricular dilatation) was described on imaging in one of two patients in the 30–40 age group. In the 40–50 age group, symptoms were still absent, but right ventricle involvement (dilation and dysfunction) and repolarization changes (inverted T waves in right precordial leads) were observed. One out of two patients had ILR, to promptly detect potential arrhythmias. In the 50–60 age group, the clinical status varied from patient to patient: one of them was asymptomatic, with no evidence on EKG and echocardiography, while the other two patients presented, respectively, late potentials on signal-averaged EKG and RV dilation. After the sixth decade of life, ACM clinical expressions became more pronounced. Starting from the 60–70 age group, all patients had palpitations and RV morphofunctional modifications. The patients of the 60–70 age group developed depolarization defects (ε waves) and biventricular affection with abnormal septal motion. In the 70–80 age group, the phenotype was fully manifested: patients became symptomatic, with the onset of arrhythmic events and imaging evidence of RV bulging and dyskinesia. Both these two patients underwent devices implantation, due to an increased risk of malignant arrhythmias and potential SCD.

### 4.3. Functional Characterization

**Figure 3:**
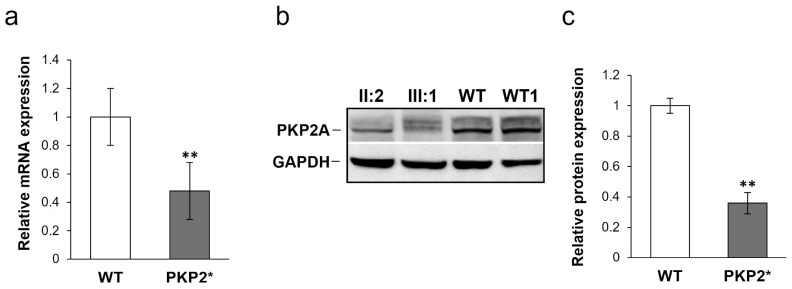
(**a**). RT-qPCR in three *PKP2* mutation carriers (PKP2* Family 1; II:2; III:1; Family 2; III:1) and two Wild-Type controls (WT). Data are from three independent experiments and are expressed in arbitrary units, normalized to the Wild-Type condition (*p*-value = 0.007). (**b**). Protein expression of PKP2. B. Representative PKP2A Western blot analysis image from WT (*n* = 2: WT1 and WT2) and *PKP2* variant carriers (PKP2* *n* = 2: II:2 and III:1). (**c**). Protein expression of PKP2 in PBMCs was quantified by densitometric analysis using WT as a unokit. Data are representative of two independent experiments and reported as mean ±SD. Mean values were compared using the two-tailed Student t-test for independent samples (** *p* < 0.01). GAPDH was used as control.

In order to verify the effect of the variant, *PKP2* gene expression was estimated by RT-qPCR in PBMCs from three *PKP2* variant carriers (Family 1; II:2; III:1; Family 2; III:1), belonging to both families and two Wild-Type (WT) controls. RT-qPCR showed that PKP2 expression levels were reduced in all three variant carriers compared with the two WT subjects. In particular, a mean reduction of 52% of PKP2 gene expression in patients carrying the variant (*p* = 0.007) was observed, suggesting that only the normal allele is expressed (Figure 3a). To find out the PKP2 expression at the protein level, the major isoform of PKP2A (92.7kDa) [15] was analyzed by Western blot (Figure 3b). As shown in Figure 3c, densitometric analysis underlines a statistically significant protein level reduction (** *p* < 0.01) in two variant carriers (Family 1; II:2; III:1) compared to WT (*n* = 2). This trend reflects the reduction of the relative gene expression obtained by RT-qPCR.

## 5. Discussion

**Figure 4:**
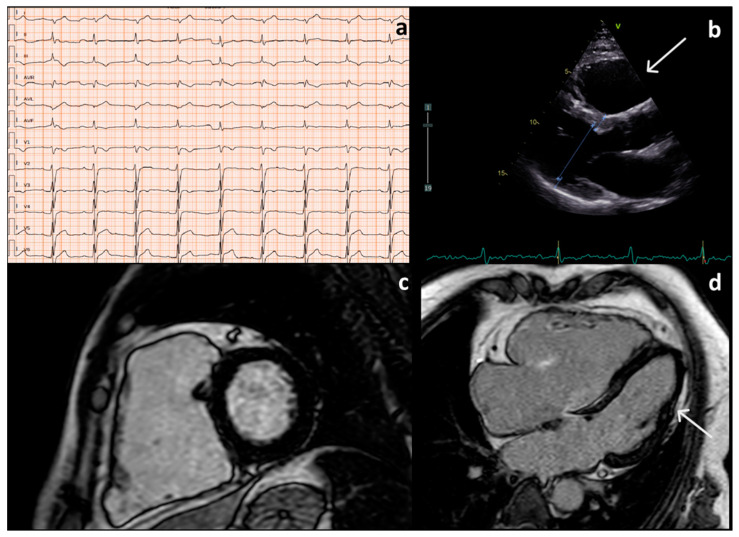
(**a**). EKG of II:2 (Family 1) shows sinus rhythm, first degree AV block, left posterior fascicular block, epsilon wave in D3, aVF and V1, and T wave inversion in D3, aVF, V3 to V4. (**b**)**.** Transthoracic echocardiography parasternal long axis 2D of II:2 (Family 1) shows right ventricle enlargement (right ventricular outflow tract dimension: 40 mm), white arrow. (**c**,**d**). Cardiac magnetic resonance imaging (CMRI) of II:2 (Family 1) reveals in short axis (**a**) severe right ventricle enlargement (right ventricle end diastolic volume180 mL/m^2^), severe dysfunction (ejection fraction 22%), and right ventricle free wall microaneurysm; the long-axis 4-chamber view (**b**) shows the presence of subepicardial late gadolinium enhancement (LGE) involving the left ventricular in the apical lateral wall (white arrow).

**Figure 5:**
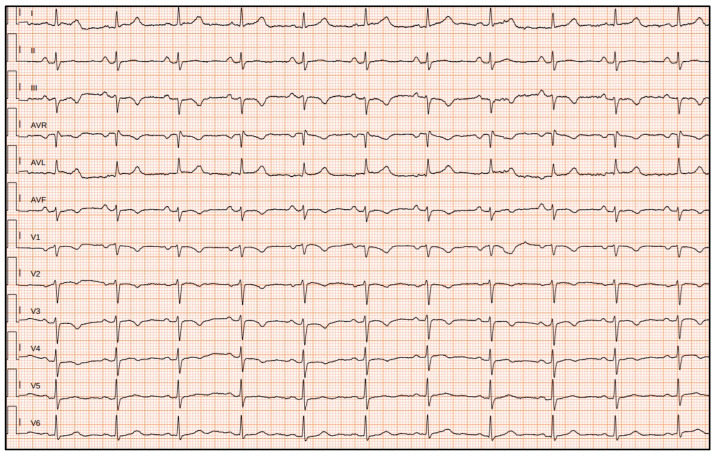
EKG of a family member (III:1, Family 2) displays sinus rhythm, left axis deviation, low QRS voltages both in the limb and precordial leads, and T-wave inversion in D3, aVF, and in V1 to V4.

Plakophilin-2 (*PKP2*) pathogenic variants are associated with ACM; however, the pathogenicity of variants within this gene is not always fully determined and no clear genotype-phenotype correlations have been described [16]. In this study, we report the molecular and clinical characterization of a heterozygous variant in *PKP2* gene, c.2443_2448delAACACCinsGAAA, detected in two different families affected by ACM. *PKP2* encodes the desmosomal armadillo repeat protein plakophilin-2, which plays a central role in linking desmosomal cadherins within the intercellular junction to intermediate filaments and the sarcomere [4]. Pathogenic variants in this gene are associated with isolated right ventricular involvement and a classic ACM phenotype [6]. The reported variant is located in the eighth and last armadillo repeat domain; it is a frameshift mutation, which introduces a premature stop codon, and consequently, produces a truncated or absent protein. This variant is very rare; indeed, it is absent in GnomAD (v.4.1) and ClinVar databases. Truncating variants represent 91.7% of pathogenic/likely pathogenic variants in *PKP2* reported in the ClinVar Database and haploinsufficiency represents the main pathogenetic mechanism underlying desmosome defects [16]. The latter finding has also been demonstrated by an ACM phenotype in patients with whole-gene deletions and heterozygous knockout mouse models [17,18]. Although cardiomyocytes and blood cells have different biological features, *PKP2* mRNA is also expressed in PBMCs and could represent an alternative and more available tissue to study variants in this gene. Remarkably, plakophilin 2 is also enriched in the karyoplasm of many cell types, including many of those lacking desmosomes [19]. Therefore, we used PBMCs as a model to investigate the expression of *PKP2* in variant carriers. Functional characterization revealed a reduction in *PKP2* gene expression in three carriers of the above-mentioned variant (Family 1; II:2; III:1; Family 2; III:1), although their clinical conditions differed in severity and expression. This phenotypic variability may be due to the effects of modifying factors, including environmental and genetic agents interacting with haploinsufficient Plakophilin 2, enough to influence the extent of signs and symptoms, age at onset, and progression of the disease [20]. Based on previously published data, it is agreed that ACM is an age-dependent condition, with a median age at presentation of 54 years in the included available patients [21]. According to this observation, the three oldest patients (Family 1, II:2 Figure 4; Family 2, III:1 Figure 5; Family 2, III:3) presented a complex and complete clinical picture characterized by specific symptomatology (palpitations and syncope), repolarization/conduction disturbances (Figure 4a and Figure 5), and morphofunctional alterations (Figure 4b–d). In proband 1 (Family 1; II:2), CMRI identified severe right ventricular enlargement and identified subepicardial late gadolinium enhancement (LGE) in the apical lateral segment of LV (Figure 4c,d).

Interestingly, III:1 and III:2 were twins, both professional athletes. At follow-up, CMRI identified in III:1 mild biventricular dilatation and LGE, whereas his twin brother did not show imaging anomalies (Table 1). Myocardial replacement by fibrous and fatty tissue is a key pathological hallmark of ACM, contributing to a myocardial decline in function and creating a substrate highly vulnerable to arrhythmias [7]. To date, many mechanisms underlying ACM remodeling remain undefined and new technologies such as transcriptomics analysis in affected tissues are disclosing new disease-driving mechanisms underlying cardiac dysfunctions. Recently, a Tomo-Seq approach (i.e., a genome-wide-transcriptomic profile with high spatial resolution) was performed on an explanted ACM heart from a *PKP2* truncating variant heterozygous carrier, revealing a *ZBTB11* expression specifically enriched in fibro-fatty replacement areas [22]. Further studies are needed to explore a possible related mechanism; however, a multi-omics approach could be a valid opportunity to improve our knowledge on pathological ACM mechanisms and develop better-targeted therapies areas [22].

Male dominance has been reported in the literature with regard to the influence of sex on penetrance, probably due to a combination of biological differences and environmental factors (such as intense physical activity or inflammatory factors) [21,23]. To corroborate this theory, a suppressive effect of estrogens on cardiomyocyte apoptosis has been reported, which may be a mitigating factor of severe and sudden onset of ACM in female *PKP2* variant carriers [24]. In line with an alleged sex-dependent penetrance, in Family 1, we reported one male patient who died of SCD at 33 years (obligate carrier, II:1) and two other male patients who manifested significant clinical features from the age of 40 years (I:1, II:2); whereas in Family 2, one male (obligate carrier) died of SCD at 67 years (III:2). Observational studies have emphasized the correlation between high-impact exercise and an increased risk of arrhythmic events (ventricular arrhythmias and/or fibrillation) in desmosomal mutation carriers [25,26]. This is the rationale behind the recommendation for the two athletes in Family 1 (III:1, III:2) to reduce the intensity and frequency of their training [25,26,27]. In fact, as recommended in the ESC Guidelines [28], avoidance of high-intensity exercise, including competitive sport, may be considered in genotype positive/phenotype-negative individuals in families with ARVC (Class II b, level of evidence C). Moreover, moderate- and/or high-intensity exercise, including competitive sport, is not recommended in individuals with ARVC (Class III, level of evidence B) [28].

Thus, genetic counseling should be also dedicated to explaining to patients that, besides the detection of a pathogenic variant, it is necessary to pay attention to secondary elements (such as lifestyle, age, gender, environment, etc.) which can impact clinical expression, increasing their awareness on adopting a balanced way of life.

In conclusion, our clinical and functional evaluation highlighted the importance of rapidly identifying individuals who are at risk of developing ACM, as the probability of manifesting malignant arrhythmias increases with age. Nevertheless, it should be noticed that the clinical onset can be sudden and severe even in young people. In view of this, paying attention to the family history allows a timely activation of a screening pathway for those who may be predisposed to cardiac events.

## Figures and Tables

**Table 1:** Clinical evaluation of *PKP2* heterozygous variant carriers based on age groups, symptoms, electrocardiogram (EKG), and imaging. Right ventricle (RV) dilatation, RV dysfunction, RV wall motion abnormalities (WMA), left ventricle (LV) dilatation, and late gadolinium enhancement (LGE) features were collected.

Fam; pt	Sex	Symptoms		ECG Findings				Morphofunctional Abnormalities				
		Palpitations	Syncope	Low QRS	ε-Waves	Negative	Negative	RV	RV	RV	LV	LGE
				Voltage		T Waves in Leads	T Waves Beyond Lead V3	Dilation	Dysfunction	Regional	Dilation	
				in Limb Leads		V1-V2/V3				WMA		
Fam 2; III:3	F	yes	/	/	yes	yes	yes	yes	yes	bulging	/	no
Fam 2; III:1	F	yes	yes	/	/	yes	yes	/	yes	bulging	/	yes
Fam 1; II:2	M	yes	/	yes	yes	/	/	yes	yes	bulging	yes	no
Fam 2; IV:7	F	/	/	yes	/	/	/	/	/	/	/	no
Fam 2; IV:6	M	/	/	/	/	/	/	/	/	/	/	no
Fam 2; IV:1	M	/	/	/	/	/	/	/	/	/	/	no
Fam 2; IV:4	M	/	/	/	/	/	/	/	/	/	/	no
Fam 2; IV:2	M	/	/	/	/	/	/	/	yes	bulging	/	no
Fam 1; III:1	M	/	/	/	/	/	/	yes	yes	/	yes	yes
Fam 1; III:2	M	/	/	/	/	/	/	/	/	/	/	no
Fam 1; III:3	M	/	/	/	/	/	yes	/	/	/	/	yes

## Data Availability

All data are available on request.

## Abbreviations

The following abbreviations are used in this manuscript:

AMI	Acute Myocardial Infarction
ACMG	American College of Medical Genetics and Genomics
ACM	Arrhythmogenic Cardiomyopathy
DCM	Dilated Cardiomyopathy
EKG	Electrocardiogram
ICD	Implantable Cardioverter Defibrillator
ILR	Implantable loop recorder
NGS	Next Generation Sequencing
PBMCs	Peripheral Blood Mononuclear Cells
PKP2	Plakophilin-2
PSVT	Paroxysmal Supraventricular Tachycardia
RV	Right Ventricle
RT-qPCR	Quantitative RT-polymerase chain reaction
SCD	Sudden Cardiac Death
WT	Wild-Type
